# Uptake, Acceptability, and Results of SARS-CoV-2 Antigen Rapid Diagnostic Testing in Community Settings in Cameroon

**DOI:** 10.4269/ajtmh.23-0802

**Published:** 2024-10-15

**Authors:** Tatiana K. Djikeussi, Boris Kevin Tchounga, Loic Feuzeu, Rogacien Kana, Boris Tchakounte Youngui, Shannon Viana, Heather J. Hoffman, Albert Mambo, Charlotte Moussi, Joseph Fokam, Emilienne Epée, Anne Hoppe, Pallavi Dani, Patrice Tchendjou, Laura Guay, Michelle M. Gill

**Affiliations:** ^1^ Elizabeth Glaser Pediatric AIDS Foundation, Yaoundé, Cameroon, and Washington, District of Columbia;; ^2^ The George Washington University Milken Institute School of Public Health, Washington, District of Columbia;; ^3^ National Public Health Emergency Operations Coordination Centre, Ministry of Public Health, Yaoundé, Cameroon;; ^4^ Virology Laboratory, Chantal BIYA International Reference Centre, Yaoundé, Cameroon;; ^5^ Faculty of Health Sciences, University of Buea, Buea, Cameroon;; ^6^ Faculty of Medicine and Biomedical Sciences, University of Yaoundé I, Yaoundé, Cameroon;; ^7^ FIND, Geneva, Switzerland

## Abstract

Mass gathering event restrictions were part of mitigation measures during the COVID-19 pandemic that were lifted as prevalence decreased and after vaccination rollout. We explored SARS-CoV-2 antigen rapid diagnostic test acceptability and positivity in community settings in Cameroon. In August–October 2022, community workers sensitized and referred individuals for COVID-19 testing to nearby testing points in Douala and Yaoundé. Participants consented to SARS-CoV-2 antigen rapid diagnostic testing, a survey, or both components. We describe the positivity rate, COVID-19–related history, and Likert-scale testing perceptions. Factors associated with testing acceptance were analyzed using logistic regression. Overall, 20.5% (2,449/11,945) of sensitized individuals visited testing points, and 1,864 (76.1%) were enrolled; 50.6% accepted the survey and testing (46.0% accepted survey only). Seven (0.7%) of 1,006 individuals tested positive. Most (71.8%; 1,292/1,800) considered community testing more accessible than hospital-based testing. Individuals accepting versus refusing testing differed in perceived COVID-19 risk (67%, 49%; *P* <0.001), belief in accurate test results (79%, 47%; *P* <0.001), and ability to test easily (96%, 55%; *P* <0.001). Males (adjusted odds ratio [aOR]: 1.26 [1.04–1.53]) and those over 50 years (aOR: 1.9 [1.4–2.7]), with symptoms (aOR: 1.80 [1.30–2.50]), and at least partial vaccination (aOR: 0.76 [0.58–0.99]) were significantly associated with test acceptance. Refusal reasons included lack of perceived need for testing (33.8%) and testing discomfort (26.3%). Although community-based testing was generally perceived as important, actual testing uptake was low. In future pandemics, community testing should be optimized by addressing misinformation, offering alternative testing modalities for greater comfort, creating demand, and tailoring approaches to maximize testing uptake.

## INTRODUCTION

Since the identification of the first cases of severe acute respiratory syndrome coronavirus 2 (SARS-CoV-2) and associated coronavirus disease-2019 (COVID-19) in 2019, the number of cases increased globally to reach 768,560,727 confirmed cases and more than 6.9 million deaths worldwide as of July 2023.^1^ In Africa, over 9.5 million cases and 175,000 deaths were reported for the same period.^1^ This likely represents a significant underestimation given the limited access to SARS-CoV-2 antigen rapid diagnostic testing (Ag-RDT), particularly outside of health systems. For most of the pandemic, SARS-CoV-2 testing in Cameroon and most of Africa focused on travelers and symptomatic individuals in hospital settings. However, many infected individuals remained asymptomatic, and in symptomatic cases, viral shedding was likely to occur before symptoms developed.^2^ This led to calls for population-level SARS-CoV-2 screening among those with and without symptoms, which required a reliable, affordable testing strategy using rapid diagnostic tests.^3^

Significant efforts were made globally to implement mitigation measures to contain the spread of SARS-CoV-2, such as lockdowns, travel restrictions, limited community gatherings, and cancellation of mass gathering events (MGEs).^4^^,^^5^ According to the WHO, mass gatherings are “events characterized by the concentration of people at a specific location for a specific purpose over a set period of time that have the potential to strain the planning and response resources of the host country or community.”^6^ A meta-analysis exploring the spread of SARS-CoV-2 during MGEs estimated a basic reproductive number (Ro) of 3.38 ± 1.40 (range: 1.90–6.49).^7^ The Ro reflects the efficiency of disease transmission; thus, in a SARS-CoV-2*–*susceptible community, a single positive case in an MGE could generate one to six secondary cases in the community. In contrast to formalized, planned events, community gathering points (CGPs) are transitory or other settings in a community typically frequented by large numbers of people at a given time (e.g., transportation hubs, markets).

The SARS-CoV-2 pandemic significantly affected the livelihood of citizens, with country lockdowns and multiple waves of infection worldwide.^8^^–^^10^ Balancing the health risks with the economic, social, and individual impacts of large national and international events, governments and event organizers had responsibility for cancelling or conducting the event, with strict measures to minimize the effects on participants and the community. There was considerable pressure to lift restrictions as SARS-CoV-2 prevalence decreased and after introduction of the COVID-19 vaccine worldwide.^11^ In addition, increased availability and convenience of testing and strengthened surveillance systems offered means to reengage in aspects of pre-pandemic life, including MGEs and large community events.

Throughout the pandemic, Cameroon’s response to limit transmission of SARS-CoV-2 focused on testing symptomatic patients within healthcare settings and travelers coming in and out of the country. The government of Cameroon decided to hold one of the largest international MGEs, the Africa Cup of Nations (AFCON) across Cameroon in January and February 2022, leading to lifting of all restrictions for MGEs and community gatherings. The COVID-19 vaccine was first available in Cameroon in April 2021, but at the time of AFCON, vaccine coverage in the general population was only 7.7% and had reached only 15% in 2023.^1^^,^^12^ With the return to unrestricted MGEs around the time of AFCON alongside large community gatherings and limited SARS-CoV-2 Ag-RDT, there was a need to better understand the extent of SARS-CoV-2 infection and acceptability of testing in broader community settings.

We conducted a study to determine the uptake, acceptability, and results of SARS-CoV-2 Ag-RDT among individuals in community settings in Douala and Yaoundé, two cities with the highest SARS-CoV-2 prevalence rates in the Littoral and Center regions of Cameroon, respectively.

## MATERIALS AND METHODS

### Study design.

In August*–*October 2022, we conducted a cross-sectional study of SARS-CoV-2 Ag-RDT and a survey on test acceptability in community settings.

### Study setting and population.

Four health districts in each city were purposively selected based on having >1,000 reported COVID-19 cases in 2022. Each district had 10 health areas, and in each health area, three high-volume venues were identified as possible points of community-based transmission. A total of 240 venues were selected for the study. Community settings included stadiums and places of worship where MGEs had been scheduled during the study period (as identified by Ministry of Health [MOH] staff and community health workers [CHWs]) and CGPs, such as markets, bus stations, and local administrative offices, with detailed cartography to guide fieldwork. The study population was composed of adults aged ≥21 years (owing to the age of consent in Cameroon) attending the selected venue where study activities were taking place.

### Study procedures.

Study data collection took place in community settings directly on the grounds of the event or within the CGP. The CHWs provided general information to individuals attending MGEs or presenting at a CGP about SARS-CoV-2 transmission and prevention and informed them of the opportunity to get tested at a nearby mobile testing point. Individuals who agreed were received at the testing site by trained research assistants who informed them about the study and the SARS-CoV-2 Ag-RDT and survey. All participants provided written informed consent for testing, the survey, or both study components. Those who declined to participate in the study or who did not meet eligibility criteria were still offered SARS-CoV-2 Ag-RDT as part of MOH routine testing activities, but their data were not included in the study analysis.

Participants were assigned a unique study identification number at enrollment that was used for study data collection and was recorded on the standard paper-based laboratory test form completed by trained MOH healthcare workers (HCWs) to link test results with survey responses. Participants were asked about sociodemographic characteristics, history of preexisting medical conditions, vaccination status, presence of COVID-like symptoms, and prevention measures taken at MGEs. Participants who accepted testing were referred to the HCWs who performed the testing on nasopharyngeal specimens using biosensor or Abbott rapid diagnostic tests (Abbott Park, IL). The HCWs also offered post-test counseling, referral for vaccination for participants who tested negative, and contact line-listing and linkage to care for those with a positive test. Those who refused testing but accepted the survey were enrolled and administered the survey. The survey captured participant’s views on topics such as SARS-CoV-2 Ag-RDT benefits, barriers to testing, trust in test results, and their risk of infection using a 5-point Likert scale. Responses to the survey were entered directly into a tablet-based data system, and SARS-CoV-2 Ag-RDT results were abstracted from laboratory records into the database.

## STATISTICAL ANALYSES

We summarized the characteristics of participants who accepted the SARS-CoV-2 Ag-RDT and survey using frequencies and percentages for categorical variables. We used medians and interquartile ranges (IQRs) to summarize age. The overall SARS-CoV-2 case detection rate was estimated as the number of SARS-CoV-2–positive tests divided by the total number of tests and expressed per 1,000 cases with the 95% CI. Factors associated with SARS-CoV-2 acceptability were explored with unadjusted and adjusted logistic regression models using a manual backward elimination approach. Odds ratios (ORs) with associated 95% CI were presented. Acceptability was explored in the groups that did and did not accept testing, with the 5-point Likert scale collapsed from five to three categorical responses (agree, no opinion, and disagree). All tests were two-sided, and the level of statistical significance was set at 0.05. Data were analyzed using Stata 17.0 (StataCorp LLC, College Station, TX).

## RESULTS

**Figure 1.**
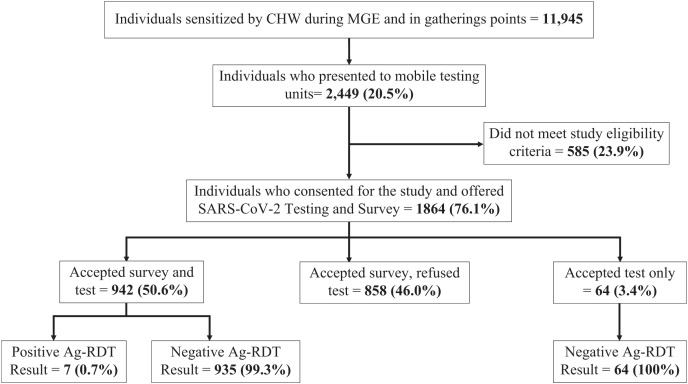
Study screening, enrollment, and Ag-RDT test results. This flow diagram presents individuals seen in community mass gathering events and gathering points, the proportion of individuals enrolled in the study, and those tested for SARS-CoV-2. Ag-RDT = antigen rapid diagnostic testing; CHW = community health worker; MGE = mass gathering event; SARS-CoV-2 = severe acute respiratory syndrome coronavirus 2.

A total of 11,945 individuals in the study communities received sensitization on SARS-CoV-2 transmission and prevention and were invited for SARS-CoV-2 Ag-RDT (Figure 1). Only 2,449 (20.5%) accessed the mobile testing units deployed for the testing campaign. Of these, 1,864 (76.1%) individuals provided informed consent and were enrolled in the study, and 585 (23.9%) did not meet eligibility criteria. The main reason for exclusion was age less than 21 years. Of those enrolled in the study, 942 (50.6%) agreed to participate in both the testing and the survey, 858 (46.0%) accepted the survey only, and 64 (3.4%) accepted testing only.

### Sociodemographic and clinical characteristics.

Overall, the median age of participants was 33 years (IQR: 26–44) with 61.1% male. Most participants (81.8%) were enrolled from CGPs and were not vaccinated or not fully vaccinated (84.8%). Among 177 (9.5%) participants who reported at least one COVID-19 sign/symptom, cough was the most common followed by runny nose and headache. A total of 121 (6.5%) participants reported at least one comorbidity, most commonly high blood pressure and diabetes.

### SARS-CoV-2 case detection rate.

Among the 1,006 (54.0%) participants who received SARS-CoV-2 Ag-RDT, seven tested positive, giving a case detection rate of 7 per 1,000 tests performed (95% CI: 2.8–14.3). Five individuals were asymptomatic, four reported that they attended an MGE in the prior 2 weeks, and none received the SARS-CoV-2 vaccine.

### Factors associated with acceptance of SARS-CoV-2 testing.

In the final logistic regression model, factors associated with testing acceptance were older age (*P <*0.0001), male sex (*P =* 0.017), testing during an MGE (*P =* 0.010), being in the Littoral region (*P <*0.0001), the presence of ≥1 COVID-like symptom (*P =* 0.0005), and being fully vaccinated (*P =* 0.044) (Table 1). Attendance at a recent MGE with or without consistent mask wearing, having a comorbidity, and previous COVID-19 illness were not significantly associated with SARS-CoV-2 Ag-RDT acceptance.

**Table 1:** Factors associated with acceptance of SARS-CoV-2 Ag-RDT

Characteristics	Accepted Testing *n* (%) *N =* 1,006	Refused Testing *n* (%) *N =* 858	Unadjusted Analysis OR (95% CI)	*P*-Value	Adjusted Analysis aOR (95% CI)	*P*-Value
Age						
Median (IQR)	35 (26–47)	32 (25–41)	–	<0.0001	–	<0.0001
≤24 Years	189 (18.8)	183 (21.3)	0.49 (0.36–0.66)		0.51 (0.37–0.70)	
25–49 Years	602 (59.8)	574 (66.9)	0.49 (0.38–0.64)		0.51 (0.39–0.66)	
≥50 Years	215 (21.4)	101 (11.8)	Ref		Ref	
Sex, *n* (%)						
Male	640 (63.6)	499 (58.2)	1.26 (1.04–1.52)	0.016	1.26 (1.04–1.53)	0.017
Female	366 (36.4)	359 (41.8)	Ref		Ref	
Testing Point						
Mass Gathering Event	204 (20.3)	135 (15.7)	1.36 (1.07–1.73)	0.011	1.40 (1.08–1.82)	0.010
Community Gathering Points	802 (79.7)	723 (84.3)	Ref		Ref	
Region						
Center	475 (47.2)	487 (56.8)	0.68 (0.57–0.82)	<0.0001	0.65 (0.54–0.79)	<0.0001
Littoral	531 (52.8)	371 (43.2)	Ref		Ref	
Presence of Symptoms						
At Least One	122 (12.1)	60 (7.0)	1.80 (1.31–2.49)	0.0003	1.80 (1.30–2.50)	0.0005
None	884 (87.9)	798 (93.1)	Ref		Ref	
COVID Vaccine Status						
Fully Vaccinated	171 (17.0)	112 (13.1)	Ref	0.018	Ref	0.044
Not/Partially Vaccinated	835 (83.0)	746 (86.9)	0.73 (0.57–0.95)		0.76 (0.58–0.99)	
Attendance and Mask Wearing at a Recent MGE						
Consistently	66 (6.6)	59 (6.7)	0.98 (0.67–1.41)	0.29	0.84 (0.58–1.24)	0.810
Partially	71 (7.1)	42 (4.9)	1.47 (0.99–2.20)		1.20 (0.79–1.81)	
Never	294 (29.2)	256 (29.8)	1.00 (0.81–1.23)		0.95 (0.76–1.18)	
Did Not Attend MGE	575 (57.2)	501 (58.4)	Ref		Ref	
Comorbidity						
At Least One	75 (7.5)	46 (5.4)	Ref	0.067	–	–
None	929 (92.5)	812 (94.6)	1.42 (0.97–2.08)		–	–
Missing	2	0	–		–	–
History of COVID-19 Disease						
Previous Infection	44 (4.4)	38 (4.4)	0.98 (0.63–1.53)	0.954	–	–
No Previous Infection	962 (95.6)	820 (95.6)	Ref		–	–

Ag-RDT = antigen rapid diagnostic testing; aOR = adjusted odds ratio; COVID-19 = coronavirus disease-2019; IQR = interquartile range; MGE = mass gathering event; OR = odds ratio; Ref = reference; SARS-CoV-2 = severe acute respiratory syndrome coronavirus 2

### Acceptability of SARS-CoV-2 Ag-RDT in a community setting.

**Figure 2.**
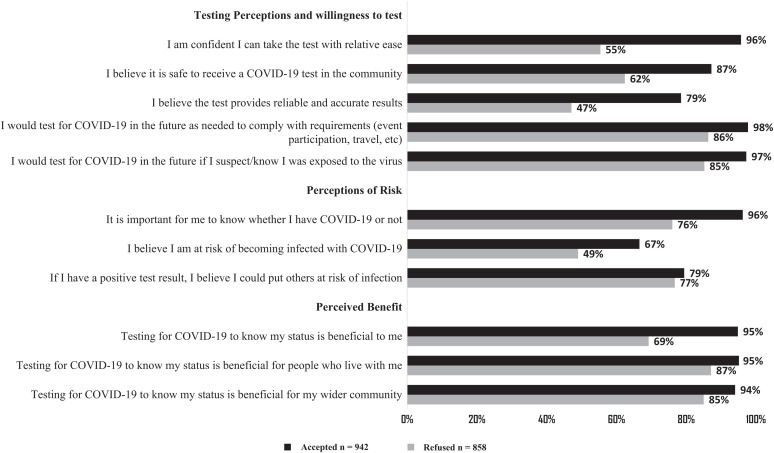
Proportion of participants who agreed with testing acceptability statements. The *x* axis represents the percentage of persons who agreed to a specific statement; the *y* axis represents the different statements grouped in three categories (willingness to test, perceptions of risk, and perceived benefit). Black bars represent those who accepted the SARS-CoV-2 rapid diagnostic test; gray bars represent those who refused the test. COVID-19 = coronavirus disease-2019; SARS-CoV-2 = severe acute respiratory syndrome coronavirus 2.

More participants who completed the survey and agreed to be tested believed that they could easily take the test (96% versus 55%; *P <*0.001) and that test results were accurate (79% versus 47%; *P <*0.001) compared with those not tested (Figure 2). Most participants in both groups agreed that they would adhere to testing requirements for events or travel or if they suspected they were exposed to the virus. Personal perception of COVID-19 risk was higher among those accepting than among those refusing testing (67% versus 49%, respectively; *P <*0.001). Participants who tested were also more likely to agree with statements on the positive benefits of testing than those not tested. The most common explanation offered among 241 participants who disagreed with the statement “I believe the test provides reliable and accurate results” was that they had heard or read information to that effect (*n =* 117, 48.5%). Others responded that COVID-19 was not real, or it was exaggerated (*n =* 102, 42.3%). Participants who refused testing were more likely to report that knowing their status was beneficial for those they live with (87%) and for their communities (85%) than it was for themselves (69%).

Most respondents who accepted testing indicated that it was because they wanted to know the result (86.9%) (Table 2). Among the 858 individuals who refused the SARS-CoV-2 test, the most common explanations were that they did not perceive a need for testing (33.8%) and reported discomfort with the test (26.3%). When asked about ways to improve the SARS-CoV-2 Ag-RDT process, the most common suggestions were to offer the test free of charge (879, 55.9%), to offer it closer to where one lives (640, 40.7%), and to reduce waiting times at testing locations (592, 37.6%). SARS-CoV-2 Ag-RDT in communities was felt to be more accessible than in hospital settings by 1,292 (71.8%) participants.

**Table 2:** Reasons for accepting or refusing SARS-CoV-2 testing

Modality	Response, *n* (%)
Reason for Accepting Testing*	*N =* 940
Just Want to Know Result	817 (86.9)
Because It Is Part of a Study	210 (22.3)
Possible Exposure	96 (10.2)
Upcoming Travel	46 (4.9)
Feel Sick	35 (3.7)
It Is an Opportunity to Test/Free of Charge	27 (2.9)
Because It Is Important to Test	13 (1.4)
Live with High-Risk Person	11 (1.2)
Other	15 (1.6)
Missing	2
Reasons for Refusing Testing	*N =* 858
Do Not Think I’m Positive/No Need	290 (33.8)
Discomfort with Test	226 (26.3)
Do Not Have Time/Too Busy	140 (16.3)
Do Not Trust/Believe the Results	63 (7.3)
Recently Tested	52 (6.1)
Fear Due to COVID-19 Misinformation	26 (3.0)
Do Not Believe COVID-19 Is Real	21 (2.4)
Other	40 (4.7)

COVID-19 = coronavirus disease-2019; SARS-CoV-2 = severe acute respiratory syndrome coronavirus 2.

* Participants could select more than one response.

## DISCUSSION

We found that most community members surveyed believed that expanding access to SARS-CoV-2 rapid testing outside of facilities through community-based testing was important, but actual testing uptake was low. Fewer than a quarter of individuals reached in study community settings presented at mobile testing services. Only half of those enrolled in the study accepted SARS-CoV-2 rapid testing, with a case detection rate of 0.7%. The low SARS-CoV-2 positivity rate found in study community settings was concordant with low national SARS-CoV-2 incidence at the time of data collection in late 2022. At this time, the “fifth wave” of COVID-19 in Cameroon was waning, about 8–10 months after the Omicron variant began circulating in the country in January 2022.^13^^,^^14^ In addition, many of the travel and gathering restrictions had been lifted, likely decreasing the perceived urgency for testing, and only individuals 21 years of age or older were included in the study.

Nonetheless, there are several lessons learned from this study that are important to inform the preparation and response to this and other emerging infectious disease threats. The ability of country health systems to rapidly respond and limit the spread of new infectious disease outbreaks depends on laboratory capacity to diagnose infections and data systems to track these infections.^15^^–^^17^ This will provide more accurate case counts and provide a more complete picture of transmission and the epidemiology of infection in the country.

Given that significant SARS-CoV-2 transmissions occurred in individuals with asymptomatic infection and that in many African countries access to testing was limited to facility-based testing of symptomatic individuals, national SARS-CoV-2 reporting significantly underestimated the extent of the pandemic.^3^^,^^15^^,^^18^ In a population-based community SARS-CoV-2 survey conducted during the “first wave” of COVID-19 in Zambia, fewer than a quarter of individuals were found in community high-volume venues.^17^ Among participants who refused community-based testing for COVID-19, there were relatively high levels of agreement regarding perceived benefits, though they reported low perceived risk and discomfort with testing.

More than 2 years into the COVID-19 pandemic, some participants in the study reported that the study testing was their first time being tested. Similarly, testing in many African countries had multidimensional challenges, among which is the accessibility of SARS-CoV-2 testing, which is mostly available in hospital settings.^19^ In the Zambia survey, few individuals seropositive for SARS-CoV-2 were aware they had been infected because they had not been tested; there was only one laboratory-confirmed case for every 92 SARS-CoV-2 infections.^17^ Fewer than 10% of individuals found to be positive in the community were aware of their infection status and 24% reported symptoms, similar to our results with only two of seven individuals testing positive in the community who reported having symptoms. These results highlight the need for rapid increase in the availability of diagnostic supplies and early expansion of testing outside of health facilities to include those without clinical symptoms. Although testing capacity has increased substantially since SARS-CoV-2 first appeared, testing volumes are still insufficient in many countries. Fortunately, there are efforts ongoing by governments to expand the availability of test kits, including through the Access to COVID-19 Tools Accelerator.^20^

Several strategies for reaching populations with limited access to health services or those in settings where increased transmission is likely to occur have been evaluated in addition to our study. Some of these include community testing points, testing in university settings or refugee camps, mass screenings, and door-to-door campaigns.^3^^,^^21^^–^^23^ Our data suggest that community testing could be a useful strategy to reach those who have been missed, often because of few interactions with the health system, by traditional, health facility–based testing, such as men and those over 50 years old. Similarly, Chugg et al.^22^ found that door-to-door testing using CHWs in areas of high prevalence was associated with a substantial increase in the proportion of Latinx and elderly individuals undergoing testing relative to neighborhood testing sites, helping to address testing equity gaps. We chose large events and meeting places in an attempt to efficiently reach large numbers; however, we found that people may be more receptive to testing if access points are within the communities where they live rather than on-site at events or places where people are busy and testing could be disruptive to daily routines. Where to focus community testing strategies during infectious disease outbreaks will require balancing the lower costs of testing in large population groups, but potentially missing the most vulnerable populations, with lower volume and potentially more cost- and staffing-intensive strategies such as community door-to-door testing.

We found that most participants acknowledged the benefits of SARS-CoV-2 Ag-RDT for themselves, family members, and the community, similar to results from studies conducted in other community settings.^22^^–^^24^ However, in our study, the biggest differences between individuals accepting and rejecting testing were a lack of perceived need to be tested, discomfort with the testing process, and lack of trust in the reliability and accuracy of test results. Similarly, lack of understanding of testing, fear of the test due to pain or possible receipt of positive results, denial of COVID-19 disease or disbelief in its severity, and lack of perceived risk were reasons for testing refusal cited in other community-based studies in Bangladesh and Brazzaville.^21^^,^^24^ Widespread misinformation in the community and financial and social consequences of testing positive are important factors that contribute to refusals. Clear, consistent, and tailored messaging around the clinical spectrum of COVID-19 disease, risk factors for both acquiring the infection and experiencing severe disease, and infection prevention behaviors, including vaccination and COVID-19 risk mitigation, are key to increasing accuracy of community knowledge and could be helpful in confronting denial of SARS-CoV-2 transmission and COVID-19 disease. Addressing these issues at the start of the next infectious disease outbreak will contribute to more rapid control of the spread of the disease.

Sensitization of communities on the benefits of SARS-CoV-2 Ag-RDT and reducing barriers to testing are critical to achieve epidemic control and reduce health disparities. Our study and others found that concern about the pain and discomfort of the collection of respiratory samples for testing also contributed to refusal to test.^21^^,^^22^ The populations are less familiar with the sampling procedures, particularly with nasopharyngeal sampling that was used in our study, and the skills needed for specimen collection require additional training that may not be feasible for large-scale testing in all settings. Alternative sampling techniques, such as nasal swabs, which have been found to be more tolerable, accessible, and easily implemented, may increase testing acceptability.^25^ The ability to collect nasal swab specimens has led to the development of simple self-testing solutions that are less uncomfortable and may be more effective for increasing SARS-CoV-2 testing, particularly for asymptomatic but high-risk individuals in rural areas or in high-burden settings.^23^^,^^26^^,^^27^ Equitable solutions for low- and middle-income settings that allow for rapid accessibility to new technologies is critical. For example, during the SARS-CoV-2 pandemic, self-testing was available at no cost throughout settings like the United States, whereas few locations in Africa had access to such testing. This will require innovation and flexibility, given the need to balance financial limitations, supply chain constraints, and structural health access limitations.^19^ Such solutions should also be culturally appropriate and tailored to local contexts.

Participants reported that in the 2 weeks prior to the survey they attended an MGE with limited compliance with infection prevention measures. Few events instituted testing or vaccination requirements in line with current national guidelines, and mask wearing was not mandatory. Seven in 10 participants reported never or almost never wearing masks or using hand sanitizer at previous MGEs. Maintaining infection prevention control is essential for minimizing community impact of infectious diseases.

Community education can play a crucial role in preparing for mass testing interventions. It helps raise awareness about the importance of testing and its benefits and provides an opportunity to properly dispel myths and misconceptions related to testing procedures, accuracy, and potential risks with clear and open communication.^28^^,^^29^ Although most participants agreed with the benefits of SARS-CoV-2 Ag-RDT and felt testing was safe, participants’ perceptions of risk of becoming infected with COVID-19 were relatively low. Tailored messaging around preventive behaviors and COVID-19 risk awareness could be helpful in addressing low perceptions of risk and denial of SARS-CoV-2 transmission and COVID-19 disease. Adherence to prevention measures at times of decreasing perception of risk is a critical area of investigation going forward. In our study, sensitizing communities about testing locations, schedules, and procedures could have ensured smoother implementation and more effectively addressed barriers (e.g., transportation, language) to testing.

This study has some limitations. Of the nearly 12,000 people reached with COVID-19 SARS-CoV-2 messaging, 80% did not agree to accompany the study team to mobile testing points. We did not capture reasons for refusal at this stage, as recruitment took place in busy settings where community members were carrying out their daily lives or enjoying an event with family or friends. Time could have played a significant factor rather than, or in addition to, acceptability of the test itself. Secondly, given the low positivity rate number of attendees with a positive SARS-CoV-2 test in the study, we were not able to perform logistic regression to understand factors associated with positive SARS-CoV-2 results.

Targeted and timely testing within the community is an important component of the diagnostic and prevention toolkit to effectively control transmission of rapidly spreading new SARS-CoV-2 variants and new emerging infections. This study adds to the global understanding of community-level motivations for testing in the SARS-CoV-2 pandemic that can inform and improve subsequent efforts. True engagement with communities to establish effective and culturally appropriate strategies that leverage on existing community infrastructure and drive better integration with facility and national structures in advance of the next infectious disease outbreak should be a priority.^30^^,^^31^ Our study points to the need for alternative options for testing outside of clinical settings for diagnosis and disease surveillance. Planning is critical to ensure that appropriate human resources and quality assurance systems are placed to allow national governments to make data-informed decisions on the control of new or emerging infectious disease outbreaks in a timely fashion.
